# Strengthening vaccine effectiveness research to rebuild public trust: A call for global participation

**DOI:** 10.1371/journal.pgph.0005359

**Published:** 2025-10-29

**Authors:** Giorgia Sulis, Melissa Brouwers

**Affiliations:** 1 School of Epidemiology and Public Health, Faculty of Medicine, University of Ottawa, Ottawa, Canada; 2 Methodological and Implementation Research Program, Ottawa Hospital Research Institute, Ottawa, Canada; PLOS: Public Library of Science, UNITED STATES OF AMERICA

Vaccine effectiveness (VE) studies are foundational to public health decision-making, especially in low- and middle-income countries (LMICs) where randomized trials are less common and real-world observational data often serve as the primary evidence base for immunization decisions [1]. Recent research has underscored the importance of exploring and characterizing public trust in vaccines, acknowledging that trust is shaped by a complex interplay of social, cultural, political, and scientific factors [2–6]. One critical influence is the credibility, rigor, and transparency of the research that informs vaccine recommendations. The science that informs vaccination policies can be difficult for the general public to interpret, especially when findings evolve rapidly or are marked by uncertainty due to evolving evidence, changing epidemiological contexts, and limitations in study design or data quality. Broadly ranging VE estimates emerging from studies conducted in different contexts and populations – sometimes without proper adjustments or acknowledgement of unique sources of bias – further contribute to uncertainty. In such contexts, clear, transparent, and methodologically sound VE research, along with transparent reporting of that research, is essential not only for guiding policy but also for fostering public understanding and confidence in the decisions that follow. As scientists, we need to do better.

Observational VE studies, which dominate the post-implementation landscape, are inherently vulnerable to bias [7–11]. These biases – ranging from confounding and selection bias to misclassification and time-varying exposures – can distort VE estimates and undermine the reliability of findings. For example, confounding by indication may occur when individuals at higher risk of disease are more likely to be vaccinated, potentially inflating or deflating VE estimates if not properly adjusted for. Similarly, misclassification of vaccination status or outcomes can lead to biased results, especially in settings with incomplete or inconsistent data. Despite their widespread use in policy and systematic reviews, VE studies are not appraised with tools tailored and operationalized to their unique methodological challenges. Existing risk-of-bias (RoB) tools, such as ROBINS-I [12] or the Newcastle-Ottawa Scale [13], offer general frameworks but lack the specificity needed to capture the nuances of VE research. For instance, these tools do not adequately account for biases specific to test-negative designs, which are commonly used for VE estimation and can substantially affect interpretation.

This gap has tangible consequences. Inconsistent RoB assessments across systematic reviews lead to variable interpretations of VE evidence, complicating synthesis and policy translation. Moreover, opaque reporting practices – such as insufficient detail on study design, bias mitigation strategies, or analytic choices – further erode confidence in VE findings. In an era of vaccine hesitancy and misinformation, such methodological opacity risks amplifying public skepticism, especially when VE estimates are contested or revised.

Improving the appraisal and reporting of VE studies is not merely a technical exercise – it is a public health imperative. Transparent, open access, high-quality VE research enables clearer communication of study findings, facilitates more reliable synthesis of evidence, and supports informed decision-making. Crucially, it also helps build and sustain public trust in vaccines by demonstrating that VE estimates are grounded in rigorous, transparent, and accountable science. We argue that a higher level of specification is required from RoB tools to ensure assessments are adequately conducted and to build knowledge and capacity about VE studies among the research community and users of research outputs.

To address these challenges, our team has just launched the Risk of Bias in Vaccine Effectiveness (RoB-VE) Project – a multi-phase, international initiative aimed at improving the quality, transparency, and interpretability of VE research. Funded by the Canadian Institutes of Health Research and endorsed by global partners including Cochrane and the EQUATOR Network, RoB-VE seeks to develop two key outputs: (1) a dedicated RoB assessment tool specifically designed to address the unique methodological features of VE studies, the exact nature and format of which will be determined during the course of the study by gathering insights from experts and knowledge users; and (2) a reporting guideline to support consistent and transparent communication of VE study methods and findings. These tools will be informed by existing models but adapted to the specific needs of VE research through engagement of diverse interest holders and empirical testing.

The RoB-VE toolkit will be developed through a rigorous, evidence-informed process that engages key interest holders and utilizes a mixed methods approach, including qualitative inquiry, measurement design, Delphi consensus, usability testing, and beta validation. Importantly, we are involving members of the public (e.g., patient advocates, community representatives, individuals with lived experience of vaccine decision-making) as well as policy decision-makers from immunization programs, public health agencies, and regulatory bodies. By doing so, we want to ensure that the tools are not only methodologically robust but also responsive to the needs of those who rely on VE evidence in practice. By embedding principles of equity, inclusivity, and transparency throughout the project, we aim to foster greater trust in VE research and its role in guiding immunization policy.

Central to this effort is the representation of diverse perspectives across geographies. VE studies are conducted and used globally, and their appraisal must reflect the realities and priorities of different contexts. We are committed to ensuring meaningful participation from a broad range of experts and interest holders, including those working in underrepresented settings and systems. Their insights are essential to developing tools that are relevant, applicable, and trusted across varied public health landscapes. This inclusive approach will help ensure that the RoB-VE toolkit supports VE research that is not only methodologically sound but also contextually appropriate and equitable.

We urge the global research community to prioritize methodological integrity and reporting transparency in VE studies. The RoB-VE Project offers a concrete pathway toward this goal. We invite researchers, reviewers, journal editors, and policy decision-makers – especially those from underrepresented regions – to engage with the initiative, adopt its tools once available, and contribute to a more trustworthy and impactful VE evidence base. Our aim is for the RoB-VE toolkit to be widely used in scientific publications, systematic reviews, and guideline/policy development, with findings communicated in ways that are accessible to both technical and non-technical audiences. By promoting consistent standards and clearer communications, RoB-VE has the potential to strengthen public trust in vaccine-related research and the decisions it informs.
